# The pathological behaviors and prognostic factors of Chinese and Japanese colorectal cancers from general hospitals: a comparative study of the inpatients with surgical operation

**DOI:** 10.18632/oncotarget.12228

**Published:** 2016-09-24

**Authors:** Xue-feng Yang, Ji-feng Zhang, Jun-jun Li, Shuang Zhao, Shuai Shi, Ji-cheng Wu, Lei Fang, Hua-mao Jiang, Hua-chuan Zheng

**Affiliations:** ^1^ Cancer Center, Key Laboratory of Brain and Spinal Cord Injury of Liaoning Province, and Animal Center, The First Affiliated Hospital of Jinzhou Medical University, Jinzhou 121001, China; ^2^ Department of Urology, The First Affiliated Hospital of Jinzhou Medical University, Jinzhou 121001, China; ^3^ Life Science Institute of Jinzhou Medical University, Jinzhou 121001, China

**Keywords:** colorectal cancers, pathological behaviors, prognosis, China, Japan

## Abstract

Here, we collected the information of 17304 and 2014 inpatients with colorectal cancer (CRC) from general hospitals of China and Japan respectively, and analyzed microscopic and macroscopic aspects, even stratified by the age and gender. It was found that Chinese CRC patients showed younger prone, more rectal and ascending cancers, less sigmoid and transverse cancers, larger size, less invasion into lymphatic system or metastasis into lymph node, and poorer differentiation than Japanese ones (*p* < 0.05). TNM staging was employed as an independent factor for the prognosis of the CRC patients regardless of the country (*p* < 0.05). Female patients showed larger tumor size, easier invasion and metastasis into lymphatic system, and worse differentiation than males (*p* < 0.05). The younger patients displayed frequent invasion and metastasis into lymphatic system, and poor differentiation in comparison to elder ones (*p* < 0.05). These findings demonstrated that Japanese patients seemed to have more invasion and metastasis due to standard and precise operation and pathological diagnosis accuracy. Actually, Chinese patients had more aggressive pathological characteristics and a poorer prognosis. Therefore, it is essential to establish a routine screening methodology, a standard treatment system and postoperative diagnosis protocol for the prevention and therapeutics of Chinese CRC patients, especially for female and young patients.

## INTRODUCTION

Colorectal cancer (CRC) is one of the most common malignancies, statistically accounting for approximately 1.36 million of new cases worldwide every year [1]. It is the third most common cancer behind lung and prostate cancers in males and the second most common after breast cancer in females. CRC is one of the leading causes of cancer-related death in the elderly. According to the SEER database, approximately 70% of cases are over the age of 65 years old, and about 40% of patients over 75 years old [2].

The incidence of CRC has been estimated to be 30–50 cases (per 100, 000) in northern America and Europe, and 3–7 cases (per 100, 000) in most Middle East countries [3]. Although epidemiological data show a marked variability around the world, and almost 60% of cases occur in developed countries, its overall incidence rate shows a slow but steady decrease (about 2% per year) [4]. However, the proportion of younger CRC cases (≤ 40 years) is 2–8 percent in western countries and about 15– 35 percent in the Middle East region [5]. GLOBOCAN showed that the amount of CRC was 253, 427 cases and ranked to the fifth in China, inferior to lung, gastric, liver and breast cancers. The death number of CRC was 139,416 cases and also ranked to the fifth in China, inferior to lung, liver, gastric and esophageal cancers. The incidence and mortality of CRC in male was higher than that in female [6].

As reviewed [7], physical inactivity, body and abdominal fatness, red and processed meat, and excessive alcohol consumption may be positively linked to CRC incidence. Some researchers demonstrate that the recent high CRC incidence in Japan may be explained by a genetic predisposition and diet, especially a limited consumption of rice [8]. Although some differences in clinicopathological characteristics of CRCs can be observed between Japanese and Chinese patients at a gross glance, there is a lack in data' support. Therefore, we collected too many cases of the disease in Takaoka Koseiren Hospital (Takaoka), The Affiliated Hospital of University of Toyama (Toyama), The Affiliated Hospital of Kanagawa Cancer Center (Kanagawa) of Japan, and The First Affiliated Hospital (CMU1), Shengjing Hospital (CMUS) and Tumor Hospital (CMUT) of China Medical University, The First Affiliated Hospital of Dalian Medical University (DMU) and Jinzhou Medical University (JMU). Their clinicopathological and prognostic profiles of the inpatients with surgical dissection were compared to further the understanding of CRC between China and Japan.

## RESULTS

### Clinicopathological features of CRC in Chinese and Japanese patients

**Figure 1:**
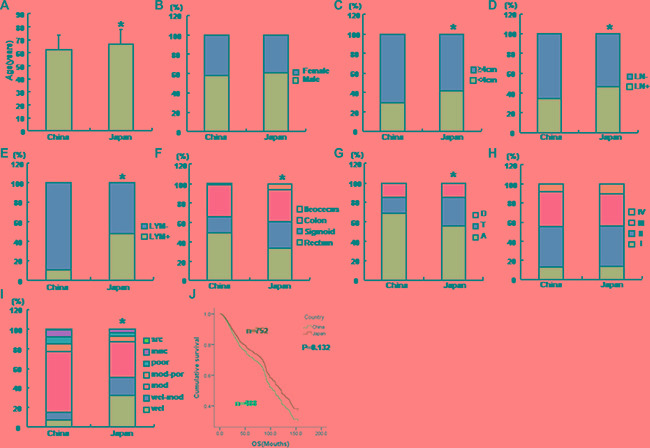
Clinicopathological features and Kaplan-Meier curves for cumulative survival rate of Chinese and Japanese patients with colorectal cancers The clinicopathological and prognostic features of Chinese and Japanese patients with colorectal cancer were compared, including age (**A**), gender (**B**), size (**C**), lymph node metastasis (**D**), lymphatic invasion (**E**), diseased parts (**F**, **G**), TNM staging (**H**), differentiation (**I**) and survival time (**J**). A, ascending colon; T, transverse colon; D, descending colon; LN, lymph node metastasis; LYM, lymphatic invasion; wel, well-differentiated adenocarcinoma; mod, moderately-differentiated adenocarcinoma; poor, poorly- differentiated adenocarcinoma; muc, mucinous adenocarcinma; src, signet ring cell carcinoma; OS, overal survival time; *p < 0.05 vs Japanese patients.

The mean age of Chinese CRC patients was 62.49 ± 11.80 (*n* = 17304), significantly younger than that of Japanese ones (Figure 1A, *p* < 0.05), which was 65.28 ± 11.11 (*n* = 2014). CRCs in Japanese patients were smaller (Figure 1C, *p* < 0.05) and more likely to invade the lymphatic system and lymph nodes than Chinese ones (Figure 1D and 1E, *p* < 0.05). Furthermore, the occurrence of rectal cancers was more common in Chinese patients, while that of sigmoid cancers less than the Japanese (Figure 1F, *p* < 0.05). Among colonic cancers, ascending cancers were frequently observed in Chinese patients in comparison to Japanese ones, while versa for transverse cancers (Figure 1G, *p* < 0.05). Histologically, Japanese CRCs exhibited good differentiation, compared to Chinese ones (Figure 1I, *p* < 0.05).

Follow-up information was available for 1240 patients with CRC for periods ranging from 0.2 months to 27 years (mean = 48.1 months). Figure 1J shows that the cumulative survival rate of Chinese CRC patients was lower than that of Japanese patients, but no significant significance was found (*p* > 0.05). Univariate analysis showed that lymph node metastasis and TNM staging were the prognostic factor for Japanese and Chinese patients with CRC (Table 1, *p* < 0.05), and related to the worse prognosis of the Chinese and Japanese patients with CRC (Table 2, *p* < 0.05). Multivariate analysis showed that TNM staging was an independent factor to indicate the prognosis of CRCs (Table 3, *p* < 0.05), regardless of the country (Table 4, *p* < 0.05).

**Table 1:** Univariate analysis of clinicopathological variables for the survival of the patients with colorectal cancer

Parameters	95.0% CI for Exp(B)	*p* value
Country	0.792 (0.584–1.073)	0.132
Sex	0.874 (0.720–1.062)	0.176
Age	0.924 (0.749–1.140)	0.461
Differentiation	1.131 (0.924–1.385)	0.232
Lymph node metastasis	1.868 (1.534–2.275)	< 0.001
TNM staging	2.668 (2.151–3.310)	< 0.001

CI = confidence interval.

**Table 2:** Univariate analysis of clinicopathological variables for the survival of Chinese and Japanese patients with colorectal cancer

Parameters	China		Japan	
	95.0% CI for Exp(B)	*p* value	95.0% CI for Exp(B)	*p* value
Sex	1.487 (0.870–2.542)	0.147	1.100 (0.892–1.356)	0.372
Age	1.258 (0.669–2.366)	0.477	1.134 (0.907–1.418)	0.269
Differentiation	2.592 (0.802–8.376)	0.112	0.930 (0.751–1.151)	0.506
Lymph node metastasis	2.937 (1.618–5.329)	< 0.001	0.577 (0.467–0.713)	< 0.001
TNM staging	3.252 (1.851–5.711)	< 0.001	0.391 (0.309–0.494)	< 0.001

CI = confidence interval.

**Table 3:** Multivariate analysis of clinicopathological variables for the survival of the patients with colorectal cancer

Parameters	95.0% CI for Exp(B)	*p* value
Country	1.210 (0.823–1.778)	0.322
Sex	1.242 (0.997–1.547)	0.053
Age	1.053 (0.817–1.310)	0.777
Differentiation	1.100 (0.872–1.387)	0.420
Lymph node metastasis	1.079 (0.804–1.448)	0.614
TNM staging	0.348 (0.255–0.474)	< 0.001

CI = confidence interval.

**Table 4:** Multivariate analysis of clinicopathological variables for the survival of Chinese and Japanese patients with colorectal cancer

Parameters	China		Japan	
	95.0% CI for Exp(B)	*p* value	95.0% CI for Exp(B)	*p* value
Sex	1.471(0.742–2.916)	0.269	1.220 (0.967–1.538)	0.094
Age	0.676(0.293–1.559)	0.358	1.061 (0.829–1.358)	0.638
Differentiation	0.406(0.096–1.720)	0.221	1.125 (0.886–1.428)	0.335
Lymph node metastasis	1.517(0.357–6.445)	0.572	1.104 (0.815–1.496)	0.523
TNM staging	0.160(0.034–0.742)	0.019	0.365 (0.266–0.502)	< 0.001

CI = confidence interval.

### The differences in clinicopathological features in the patients with CRC

**Figure 2:**
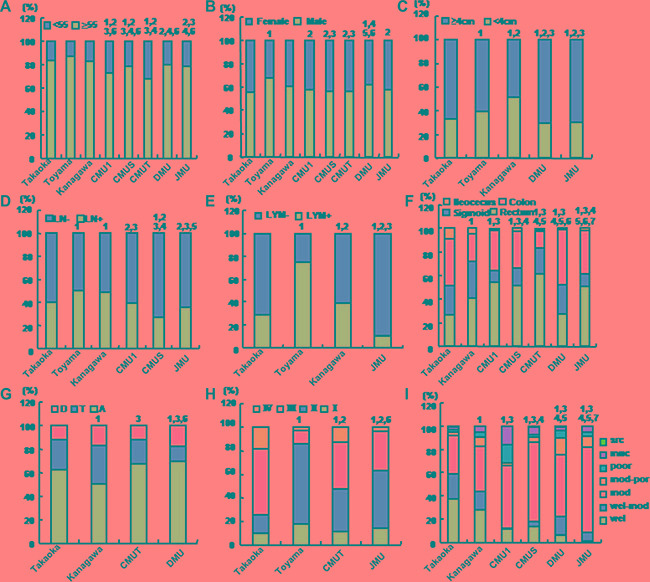
The differences in clinicopathological features of the patients with colorectal cancer from different hospitals The clinicopathological features of the patients from different hospitals were compared, including age (**A**), gender (**B**), size (**C**), lymph node metastasis (**D**), lymphatic invasion (**E**), diseasedparts (**F**, **G**), TNM staging (**H**), differentiation (**I**). Takaoka, Takaoka Kouseiren Hospital; Toyama, The Affiliated Hospital of University of Toyama; Kanagawa, The Affiliated Hospital of Kanagawa Cancer Center; CMU1, The First Affiliated Hospital of China Medical University; CMUS, Shengjing Hospital of China Medical University; CMUT, Tumor Hospital of China Medical University; DMU, The First Affiliated Hospital of Dalian Medical University; JMU, The First Affiliated Hospital of Jinzhou Medical University. A, ascending colon; T, transverse colon; D, descending colon; LN, lymph node metastasis; LYM, lymphatic invasion; wel, well-differentiated adenocarcinoma; mod: moderately-differentiated adenocarcinoma; poor, poorly- differentiated adenocarcinoma; muc, mucinous adenocarcinma; src, signet ring cell carcinoma. 1, *p* < 0.05 vs Takaoka; 2, *p* < 0.05 vs Toyama; 3, *p* < 0.05 vs Kanagawa; 4, *p* < 0.05 vs CMU1; 5, *p* < 0.05 vs CMUS; 6, *p* < 0.05 vs CMUT; 7, *p* < 0.05 vs DMU.

The patients were younger in CMU1, CMUS, and CMUT than those in Japanese three hospitals, while CMUS, DMU and JMU elder than CMU1 and CMUT (Figure 2A, *p* < 0.05). The male CRC patients were more frequently diagnosed in Toyama and DMU than Takaoka, while Toyama than CMU1, CMUS, CMUT and JMU, Kanagawa than CMUS and CMUT, and DMU than CMU1, CMUS and CMUT (Figure 2B, *p* < 0.05). The tumor size was larger in Takaoka than Toyama and Kanagawa, which was smaller than DMU and JMU (Figure 2C, *p* < 0.05). Regarding lymph node metastasis, the frequency was higher in Toyama and Kanagawa than Takaoka. Lymph node metastasis was less frequently detectable in three Chinese hospitals than Toyama and Kanagawa (Figure 2D, *p* < 0.05). Higher frequency of lymphatic invasion was observed in Toyama than Takaoka and Kanagawa, which of the three hospitals was higher than JMU (Figure 2E, *p* < 0.05). CMU1, CMUS, CMUT and JMU showed higher incidence of rectal cancers than two Japanese hospitals. DMU did lower than the other hospitals, while CMUT did higher than the other four Chinese hospitals (Figure 2F, *p* < 0.05). Most CRC occurred in ascending colon, while the rate of ascending colon from Kanagawa was lowest in ascending regions, and that from DMU highest (Figure 2G, *p* < 0.05). TNM staging of CRCs was lower in Toyama and JMU than Takaoka and CMUT, whereas that in Toyama than JMU (Figure 2H, *p* < 0.05). The tumor differentiation was higher in Takaoka than Kanagawa, where that was higher than CMU1, CMUS, DMU and JMU. In Chinese hospitals, the differentiation ranked from CMUS, DMU, JMU to CMU1 (Figure 2I, *p* < 0.05).

### Clinicopathological features of CRC patients of genders and different ages

**Figure 3:**
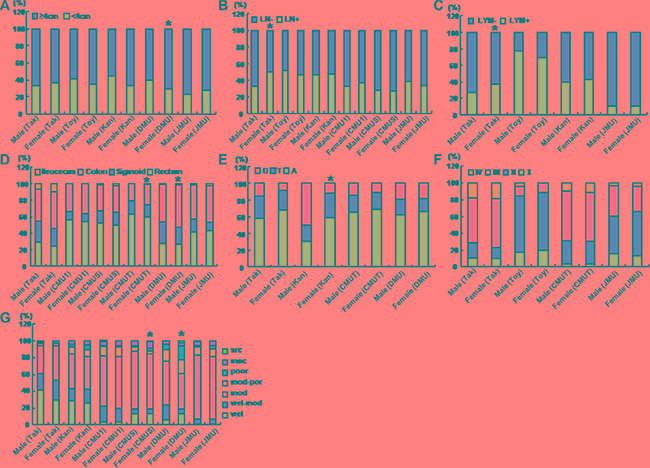
The clinicopathological features of the colorectal cancer patients of different genders According to the gender, the clinicopathological features of the patients with colorectal cancer were compared, including size (**A**), lymph node metastasis (**B**), lymphatic invasion (**C**), diseased parts (**D**, **E**), TNM staging (**F**), and differentiation (**G**) from different hospitals. Takaoka, Takaoka Kouseiren Hospital; Toyama, The Affiliated Hospital of University of Toyama; Kanagawa, The Affiliated Hospital of Kanagawa Cancer Center; CMU1, The First Affiliated Hospital of China Medical University; CMUS, Shengjing Hospital of China Medical University; CMUT, Tumor Hospital of China Medical University; DMU, The First Affiliated Hospital of Dalian Medical University; JMU, The First Affiliated Hospital of Jinzhou Medical University. A, ascending colon; T, transverse colon; D, descending colon; LN, lymph node metastasis; LYM, lymphatic invasion; wel, well-differentiated adenocarcinoma; mod, moderately-differentiated adenocarcinoma; poor, poorly- differentiated adenocarcinoma; muc, mucinous adenocarcinma; src, signet ring cell carcinoma; *p < 0.05 vs male patients.

As shown in Figure 3A, the female patients with CRC showed a larger tumor size than the male ones in DMU (*p* < 0.05). There was more lymphatic invasion and lymph node metastasis for the female than male patients in Takaoka (Figure 3B and 3C, *p* < 0.05). More colon cancers were detectable in female than male patients from CMUT and DMU (Figure 3D, *p* < 0.05). In Kanagawa, ascending colon cancers were more frequently observed in female than male patients (Figure 3E, *p* < 0.05), while versa for descending colon cancer (Figure 3E, *p* < 0.05). Histologically, female patients showed more mucinous carcinoma and less moderately-differentiated adenocarcinoma than male ones in CMUS (Figure 3G, *p* < 0.05). In DMU, more poorly-differentiated adenocarcinoma, but less well- to moderately-differentiated adenocarcinoma were diagnosed in female than male patients with CRC (Figure 3G, *p* < 0.05).

**Figure 4:**
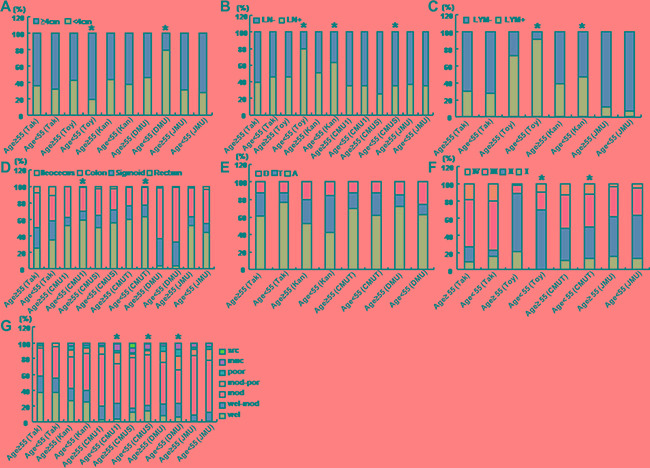
The clinicopathological features of the colorectal cancer patients of different ages According to the different age, the clinicopathological features of the patients with colorectal cancer were compared, including tumor size (**A**), lymph node metastasis (**B**), lymphatic invasion (**C**), diseased parts (**D**, **E**), TNM staging (**F**), and differentiation (**G**) from different hospitals. Takaoka, Takaoka Kouseiren Hospital; Toyama, The Affiliated Hospital of University of Toyama; Kanagawa, The Affiliated Hospital of Kanagawa Cancer Center; CMU1, The First Affiliated Hospital of China Medical University; CMUS, Shengjing Hospital of China Medical University; CMUT, Tumor Hospital of China Medical University; DMU, The First Affiliated Hospital of Dalian Medical University; JMU, The First Affiliated Hospital of Jinzhou Medical University. A, ascending colon; T, transverse colon; D, descending colon; LN, lymph node metastasis; LYM, lymphatic invasion; wel, well-differentiated adenocarcinoma; mod, moderately-differentiated adenocarcinoma; poor, poorly-differentiated adenocarcinoma; muc, mucinous adenocarcinma; src, signet ring cell carcinoma; *p < 0.05 vs elder patients (≥ 55 years).

As shown in Figure 4A, the younger patients showed a larger tumor size than the elder in Toyama, but the converse for DMU (*p* < 0.05). There was more lymph node metastasis or lymphatic invasion in the younger patients of Toyama, Kanagawa or CMUS than the elder counterpart (Figure 4B and 4C, *p* < 0.05). In CMU1 and CMUT, rectal cancers more frequently occurred in the younger than elder patients (Figure 4D, *p* < 0.05). In Toyama, colorectal cancer showed advanced TNM staging in younger than elder patients (Figure 4F, *p* < 0.05). Moderately-differentiated adenocarcinoma was less frequently observed in the younger than elder patients (Figure 4G, *p* < 0.05), while versa for mucinous adenocarcinoma (Figure 4G, *p* < 0.05). In CMUS, the elder patients more suffered from signet ring cell carcinoma than younger ones (Figure 4G, *p* < 0.05).

## DISCUSSION

Either China or Japan has experienced a double to quadruple increase in the incidence of CRC during the past few decades [9, 10]. Although the alteration in dietary habits and lifestyle is believed to account for the increase, the interaction between environmental factors and genetic characteristics might play a pivotal role in colorectal carcinogenesis of Asian populations. In Japan, large scale of beef and pork imports started after World War II and a steep rise in colorectal cancer incidence was noted after 1970. The contamination of oncogenic bovine viruses (e.g. human papillomavirus and JC virus) might be risk factors for CRC [11–13]. In line with our data, Shanghai patients with CRC were younger, notably more common with mucinous adenocarcinoma and the most frequent site was the rectum than those of Sakai group [14]. Among CRCs, ascending cancers were more frequently observed in Chinese than Japanese patients, while versa for transverse cancer. Furthermore, CRCs of Japanese patients were more likely to grow small, invade the lymphatic system, metastasize into lymph nodes and exhibit good differentiation than Chinese population. In contrast, the rate of venous or lymphatic invasion was shown to be lower in Chinese than Japanese patients with CRC. Japanese surgeons generally dissect CRCs according to the protocol of Japanese Cancer Society, including the removal of lymph nodes for detailed pathodiagnosis. Japanese pathologists generally utilize Elastic-van Gieson (EvG) and D2–40 immunostaining to characterize microinvasion, which dramatically increases the ability to identify lymphatic and venous invasion. Additionally, Japanese patients with CRC were found to have a more favorable prognosis than Chinese patients and TNM staging was employed as an independent prognosis factor for CRC patients of China and/or Japan. Therefore, it is believed that Chinese patients had more aggressive pathological characteristics and a poorer prognosis than Japanese patients although Japanese patients have more lymph nodes and lymphatic invasion metastasis. This phenomenon might be due to the following reasons: (1) Japanese health insurance system may promote early discovery and diagnosis of CRCs; (2) A systematic population- screening program of CRC has been established by utilizing sensitive and specific colonoscope.

In Japan, we selected three hospitals from Takaoka, Toyama and Kanagawa, and found that CRCs were large, localized to colon, of better differentiation and low TNM staging in Takaoka, which showed more lymphatic invasion and lymph node metastasis. Takaoka is the high-incidence area of CRC and rich in the experience of early prevention and diagnosis. In Toyama, CRCs showed frequent lymphatic invasion and lymph node metastasis, and advanced TNM staging. Toyama younger patients displayed small tumor size, frequent lymphatic invasion and lymph node metastasis. CRC cases come from the Affiliated Hospital of Toyama University, where the high medical level determines the severity of the disease and the precision of pathodiagnosis. CRCs in Kanagawa were characterized by small tumor size, the distribution of rectum, poor differentiation, frequent lymph node metastasis and lymphatic invasion. Toyama and Takaoka are localized in the developing west of Japan, while Kanagawa is in her developed east area and ranks the second. Here, the encosocial burden and mental stress are high so that the inhabitants intake too much meat, sweets and alcohol and have no enough time for health examination. Kumagai et al. [15] found that a high-dairy, high-fruit-and-vegetable, and low-alcohol dietary pattern was found to be negatively associated with the risk of colorectal cancer, but high consumption of red meat and processed meat had the positive correlation [16]. Habitual smoking increased, while frequent physical exercise and raw vegetables intake decreased colorectal cancer risk, regardless of the first-degree relative status [17].

In China, the five hospitals of Liaoning Province were enrolled in the present study, among which DMU and JMU were localized near to the sea, while CMU1, CMUS and CMUT in the provincial capital. In JMU, CRCs displayed small portion of well-differentiated adenocarcinoma, large portion of moderately-differentiated adenocarcinoma, and rectal distribution, as well as better differentiation in younger patients. Male patients and colonic localization, especially ascending colon were often observed in DMU. As for female patients, CRCs were more detected, large and poorly differentiated. In younger patients, tumor size was comparatively small and poor differentiation. Both cities are coastal, where the residents have the high intake of fresh and alcohol. Although higher fresh fish intake was inversely associated with colorectal cancer risk [18], the bad cooking approaches and industry-polluted seafoods also enhanced the intake of carcinogen. In agreement with the report of Yuan et al. [19], overdrinking also increases the carcinogenic risk of CRC when the people enjoy the marine products, especially in Jinzou, well-known for barbecue. In CMU1, there seemed more poorly- differentiated adenocarcinoma, more well- differentiated adenocarcinoma, more lymph node metastasis, more mucinous adenocarcinoma in female patients, and more lymph node metastasis in young patients. Here, CRCs are dissected and pathologically diagnosed strictly according to the guidelines of Japanese Cancer Society. In CMUS, they had a better differentiation and lower prone to invasion and metastasis in lymph node. Most of the patients were female and more common in younger patients. The female patients exhibited a poor differentiation and higher mucinous adenocarcinoma. The hospital is very famous and receives many patients so that comparatively unenough doctors cause the lack in the accuracy and precision of the treatment and subsequent pathological diagnosis, such as lymph node metastasis and lymphatic invasion. In CMUT, the patients with CRC were young and mostly distributed to rectum, while to colon for female. Regarding young patients, more cancer was localized to rectum and showed comparatively early TNM staging. The last three hospitals are localized in Shenyang, and receive many advanced and serious patients with CRC from the other cities of Liaoning Province.

Although obesity, particularly central obesity, was found to associate with an increased risk of CRCs in Chinese men [20], Sakishima found [21] that obese rectal cancer patients have high disease-free survival rates and a decreased incidence of distant metastases compared to non-obese patients. It is suggested that body weigh control can prevent CRCs and high nutrient supplement will ameliaorate the prognosis of the patients with CRC. Coffee or coffee polyphenols consumption reduced the overall occurrence of colorectal adenoma and adenocarcinoma, because coffee is a commonly consumed beverage which contains several potential anticarcinogenic and chemopreventive compounds [22–25]. Reportedly, the consumption of green tea, α-carotene, β-carotene, β-cryptoxanthin, lycopene, total n-3 polyunsaturated fat, α-linolenic acid, and long-chain n-3 polyunsaturated fat, fruit and magnesium intake were generally inversely associated with the risk of CRCs [26–30], whereas Wu et al. [31] found that circulating CRP level is positively associated with CRC risk in Chinese men. Therefore, we should guide the population to increase the intake of anti-oxidant and anti-DNA damage food and to avoid the inflammatory conditions. In addition, it is necessary to strengthen the social support and health education, and perform such CRC screening as fecal occult blood testing and colorectal endoscopy.

In summary, Japanese patients seemed to have more lymph nodes and lymphatic invasion metastasis. Actually, we found that Chinese patients had more aggressive pathological characteristics and a poorer prognosis than Japanese patients. Consequently, it is necessary to build up a routine screening methodology, a standard treatment system and postoperative diagnosis protocol for the prevention and therapeutics of Chinese CRC patients, especially for female and young patients. However, the epidemiological differences in the clinicopathological parameters of CRC between Japan and China still require further future investigation to clarify the underlying reasons behind these phenomena.

## MATERIALS AND METHODS

### Subjects

19318 CRCs were collected from surgical resection in Takaoka Koseiren Hospital (*n* = 342), The Affiliated Hospital of University of Toyama (*n* = 160) and The Affiliated Hospital of Kanagawa Cancer Center (*n* = 1512) of Japan between 1995 and 2014, and The First Affiliated Hospital (*n* = 1450), Shengjing Hospital (*n* = 2139) and Tumor Hospital (*n* = 11449) of China Medical University, The First Affiliated Hospital of Dalian Medical University (*n* = 1576) and Jinzhou Medical University (*n* = 690) between 2007 and 2014. However, incomplete clinicopathological data made a smaller number of some parameters. None of these cases underwent either chemotherapy or radiotherapy before surgery. The Ethical Committees of these hospitals approved the research protocol.

### Pathology

The staging for each colorectal cancer was evaluated according to the TNM system of the Internationale Contre le Cancer indicating the extent of tumor spread. The histomorphological architecture of the tumors was expressed according to WHO's classification. In addition, lymphatic and venous invasion, and lymph node metastasis were determined.

### Statistical analysis

SPSS 10.0 software program was employed to analyze all data. Fisher's exact possibility was performed to differentiate the rates and the Mann-Whitney *U* test to differentiate the means of the different groups. Kaplan-Meier survival plots were generated and comparisons between the survival curves were made with the log-rank statistic. Cox's proportional hazards model was employed for multivariate analysis. *P* < 0.05 was considered to represent a statistically significant difference.
